# Cluster-set rest *vs.* traditional during repeated sprint training: effects on creatine phosphokinase activity, sprint abilities and 100-m sprint performance

**DOI:** 10.7717/peerj.21689

**Published:** 2026-09-21

**Authors:** Mohamed Megahed

**Affiliations:** Department of Sports Health, College of Sports Sciences & Physical Activity, Princess Nourah bint Abdulrahman University, Riyadh, Saudi Arabia

**Keywords:** High-intensity training, CPK, Fatigue index, Athletics, Work-to-rest intervals

## Abstract

**Background:**

This study examined the effects of cluster-set rest and traditional rest interval configurations during repeated sprint training on creatine phosphokinase enzyme (CPK) activity, sprint abilities, and 100-m sprint performance.

**Methods:**

The study was carried out using a nonrandomized experimental design involving twenty well-trained male sprinters. Participants were assigned to two equally sized groups: the Cluster Set Rest Group (CSRG) and the Traditional Rest Group (TRG). The eight-week training intervention included 24 sessions, with three weekly training days. All variables were assessed pre- and post-intervention. CPK activity was assessed through biochemical analysis, while sprint abilities were evaluated using the 0–40 m sprint and repeated sprint ability 6 × 30-m (RSAT 6 × 30) tests. The 100-m sprint time (100 m SP time) was measured in accordance with the regulations set forth by world athletics.

**Results:**

Significant group × time interactions were identified using a 2 × 2 mixed-factorial Analysis of Variance (ANOVA) for CPK activity (*p* < 0.001), RSAT 6 × 30 parameters (peak sprint speed: *p* = 0.003; mean sprint speed: *p* = 0.010; fatigue index: *p* = 0.009), and 100-m SP time (*p* = 0.031), with the CSRG exhibiting significantly greater improvements than the TRG. CPK activity increased from 214.4 ± 40.45 U/L to 358.2 ± 18.74 U/L in the CSRG compared with 224.9 ± 29.03 U/L to 302.6 ± 13.84 U/L in the TRG. Likewise, 100-m SP time improved from 12.08 ± 0.25 s to 11.80 ± 0.16 s in the CSRG, whereas the TRG improved from 12.12 ± 0.24 s to 11.96 ± 0.25 s. No significant group × time interactions were detected during the acceleration phases (0–10 m, 0–20 m, and 0–30 m) of the 0–40 m sprint test (*p* = 0.160, 0.235, and 0.265, respectively). In contrast, a significant interaction effect was identified during the flying sprint phase (20–40 m; *p* = 0.008).

**Discussion:**

These findings highlight the efficacy of cluster-set rest (CSR) protocols in enhancing energy-system function and sprint performance, providing practical implications for the design of high-intensity sprint-training programs. These results may assist coaches and athletes in designing sprint-training programs that optimize recovery distribution and performance outcomes.

## Introduction

The 100-m sprint is a key event in athletics, classified within sprint events (which cover distances from 100 to 400 m) and distinguished by its greater intensity compared to middle- and long-distance running (Freeman, 2015). The overarching objective in sprint events is to execute motor actions with minimum time and maximum efficiency. Achieving this in the 100-m sprint necessitates that athletes focus on attaining and maintaining maximum sprint velocity until crossing the finish line (Thompson, Müller & Ritzdorf, 2009). Traditionally, the 100-m sprint event is divided into distinct phases: reaction at the start, acceleration, maximum sprint velocity, deceleration, and the finish phase (Wibowo, Sidik & Hendrayana, 2017). The maximum sprint velocity and deceleration phases are collectively referred to as the speed maintenance phase (Chatzilazaridis, Panoutsakopoulos & Papaiakovou, 2012; Hunter, Marshall & McNair, 2004). Successful performance in this event depends on an athlete’s ability to excel in each phase, necessitating the development of sprinting abilities that combine acceleration, maximum sprint velocity, and fatigue resistance (Debaere, Jonkers & Delecluse, 2013). Therefore, structured and targeted training is essential to enhance the sprinting capabilities necessary for optimizing performance in this event (Raj & Maniazhagu, 2022; Wang & Wang, 2023). Performance in the 100-m sprint is predominantly governed by the anaerobic energy systems, particularly the phosphagen system, which provides the primary energy source for high-intensity efforts lasting between 6 and 10 s (Amneus et al., 2012; Rogers, 2000).

Training protocols designed to enhance anaerobic performance typically emphasize adaptations in energy system capacity, muscle fiber recruitment, and metabolic efficiency (Freeman, 2015). Among these, repeated sprint training (RST) has emerged as a cornerstone for developing sprint abilities, as it involves multiple short-duration sprints separated by recovery periods, imposing high demands on both neuromuscular and metabolic systems (Beard et al., 2019; Buchheit et al., 2010; Buchheit & Laursen, 2013; Girard, Mendez-Villanueva & Bishop, 2011). This training approach contributes to increased activity of creatine phosphokinase (CPK), a key anaerobic enzyme that facilitates rapid Adenosine Triphosphate (ATP) resynthesis through the phosphagen energy system during high-intensity sprint efforts (Kaczor et al., 2005; Spencer et al., 2005). Previous literature has also indicated that manipulating rest intervals during sprint training may influence CPK activity and, consequently, affect athletic performance (Cadefau et al., 1990; MacDougall et al., 1998). Additionally, RST has been shown to improve specific sprinting metrics, including maximum sprint velocity and repeated sprint ability (Bishop, Girard & Mendez-Villanueva, 2011; Girard, Mendez-Villanueva & Bishop, 2011; Spencer et al., 2005). Given the importance of both metabolic adaptations and sprint-performance outcomes, and because recovery from high-intensity sprint efforts is strongly influenced by the duration and distribution of rest intervals, recovery structure has become a key variable in sprint-training design.

Among the adjustable variables in RST protocols, the duration and structure of recovery periods play a pivotal role in determining training outcomes (Bogdanis et al., 1995; Ohya, Aramaki & Kitagawa, 2013). Traditional recovery protocols typically involve uniform rest intervals between sprints, allowing athletes to recover sufficiently to perform subsequent efforts at maximum or near-maximum intensity (Nikolaidis & Knechtle, 2016). However, longer recovery periods may reduce the metabolic stimulus associated with anaerobic training adaptations. Conversely, shorter recovery intervals, while imposing greater physiological stress, may lead to excessive fatigue, potentially compromising sprint quality and increasing the risk of overtraining (Ratel et al., 2002). Consequently, considerable attention has been directed toward identifying recovery strategies that can optimize training quality while minimizing fatigue accumulation.

Cluster-set rest (CSR) is an innovative approach to modifying recovery periods in RST. CSR involves dividing a series of sprints into smaller subsets with brief intra-set rest periods. This strategy aims to balance fatigue accumulation with recovery, enabling athletes to maintain higher sprint quality throughout the training session (Moghadam et al., 2023; Tufano et al., 2018). Studies suggest that CSR can mitigate performance declines associated with neuromuscular fatigue, as evidenced by reduced increases in electromyographic amplitude and preserved frequency characteristics (Ortega-Becerra, Sánchez-Moreno & Pareja-Blanco, 2021). Additionally, CSR has been associated with better performance maintenance and less reduction in power and velocity compared to traditional recovery protocols, suggesting a lower accumulation of training-induced fatigue (Haff & Harden, 2022; Páez-Maldonado et al., 2024; Tufano et al., 2016; Tufano et al., 2018).

Despite its theoretical advantages, evidence regarding the application of CSR in the context of RST for 100-m sprint performance remains limited. Research on CSR has primarily focused on resistance training or plyometric training, with relatively few studies examining its effects on sprint-specific performance variables such as peak velocity, average power output, and metabolic recovery dynamics (García-Ramos et al., 2015; Moghadam et al., 2023; Nikolaidis & Knechtle, 2016). Furthermore, the role of CSR in modulating anaerobic enzyme activity, a critical determinant of sprint performance, has received limited attention. Given the importance of optimizing recovery strategies to enhance sprint performance, it is essential to compare the physiological and performance adaptations resulting from traditional recovery strategies and CSR.

This study aimed to examine the effects of CSR compared with traditional rest intervals during RST on CPK activity, sprinting abilities, and 100-m sprint performance. By exploring the physiological and performance adaptations associated with these recovery strategies, the study sought to provide practical, evidence-based insights that may help athletes and coaches optimize sprint training and enhance competitive performance. It was hypothesized that CSR would enhance sprinting abilities and 100-m sprint performance while eliciting distinct changes in CPK activity compared with traditional rest intervals during repeated sprint training.

## Material and Methods

### Participants

Twenty male sprinters were recruited for participation in this study from the department of track and field at the faculty of sports science, Arish University. All participants were experienced athletes with prior specialized training in sprint events. They were allocated into two groups of equal size: the Cluster Set Rest Group (CSRG) and the Traditional Rest Group (TRG). Eligibility criteria for inclusion in the study were as follows: (i) demonstrated technical competence and proficiency in sprinting disciplines; (ii) demonstrated commitment to completing the training program; and (iii) absence of any preexisting medical conditions or physical impairments that could influence the study outcomes. Before participation, athletes were provided with comprehensive information about the study aims, testing procedures, and participation requirements. All athletes provided written consent prior to participation and data collection, in compliance with institutional guidelines. Ethical clearance for the study was granted by the Arish University Research Ethics Committee (Reference No. ARU052), and the investigation was conducted in accordance with recognized ethical standards for studies involving human volunteers. Baseline characteristics of the two groups, including demographic and performance-related variables, were assessed to confirm group comparability and homogeneity. A summary of participant characteristics is presented in Table 1.

**Table 1:** Baseline characteristics of the study participants and between-group comparison results (mean ± SD).

	**CSRG (*n* = 10)**		**TRG (*n* = 10)**		***T***	***P***	**Sig**
	**Mean ± SD**	**CV**	**Mean ± SD**	**CV**			
Height (cm)	179.4 ± 7.52	4.19	178.3 ± 6.11	3.43	0.359	0.268	NO
Body mass (kg)	74.3 ± 5.38	7.24	69.7 ± 2.34	3.39	2.178	0.056	NO
Age (year)	21.8 ± 0.79	4.00	21.5 ± 0.53	2.45	1.238	0.192	NO

**Notes.**

CSRG cluster set rest group TRG Traditional Rest Group *n* sample size *P* *P*-value CV Coefficient of Variation NS No statistical difference between groups

### Study design

A nonrandomized trial was conducted to examine the effects of an intervention across two groups: CSRG and a TRG. The study employed a baseline—post-test design and was organized into four distinct phases: (i) Preparatory Phase (1 week): This phase familiarized participants with the study’s assessments and training protocols. The reliability and validity of all tests and tools were verified during this period. Demographic and baseline characteristics were collected as part of the familiarization process. Participants then proceeded to the baseline testing phase. (ii) Baseline Testing Phase (1 week): Pre-intervention assessments were conducted to establish baseline performance metrics for all participants. This phase adhered to standardized testing protocols to ensure accuracy and consistency. Participants then entered the training phase according to their assigned group. (iii) Training Phase (8 weeks): Participants completed an eight-week training program designed according to their assigned group. The CSRG followed a CSR protocol, while the TRG adhered to traditional rest intervals. Following the intervention period, post-intervention assessments were conducted. (iv) Post-testing Phase (1 week): Post-intervention assessments were conducted using the same protocols as in the baseline phase to ensure consistency and comparability of results.

### Testing procedures

**Figure 1:**
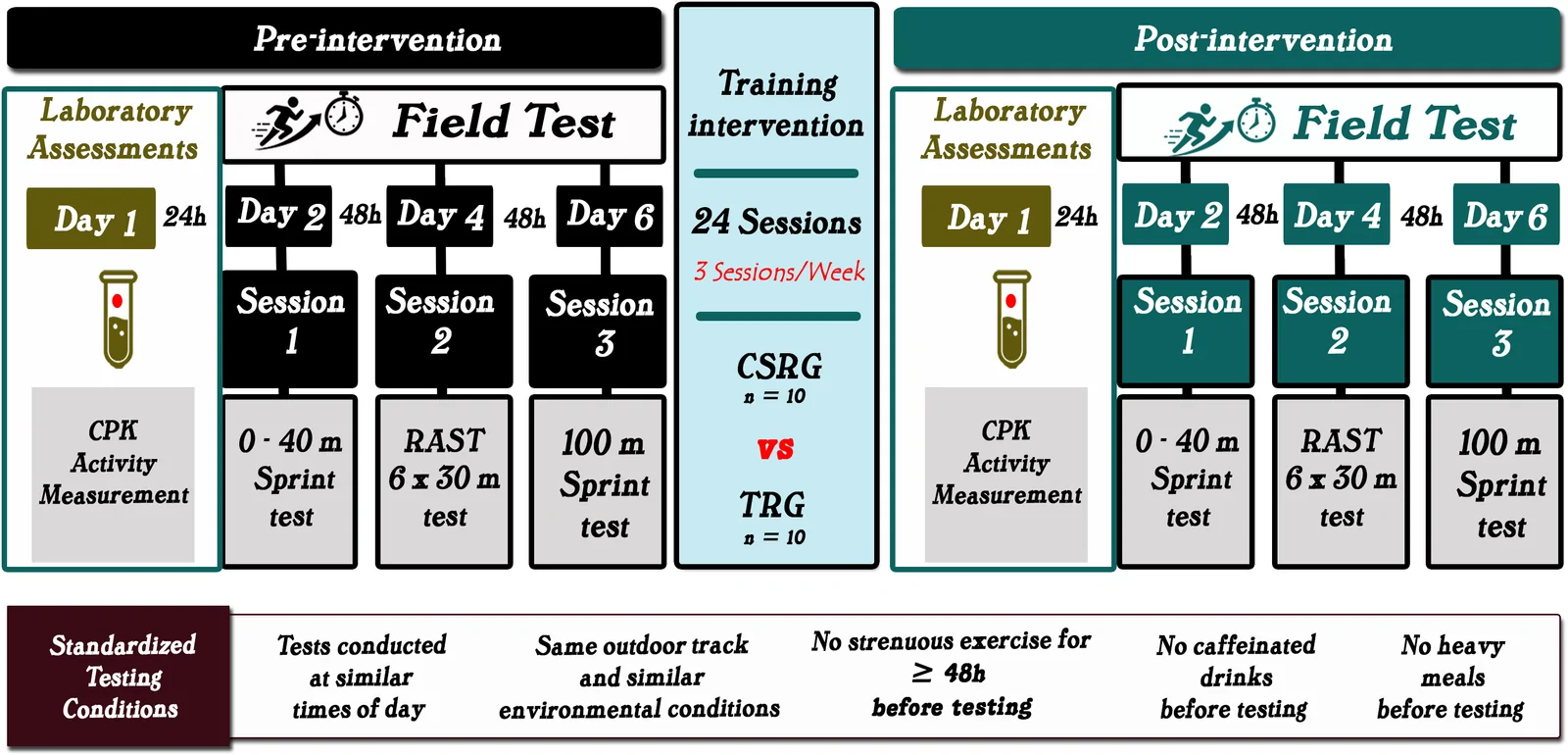
Timeline of pre- and post-intervention testing procedures.

Participants underwent evaluations in two distinct settings: the laboratory and the field, with a 24-hour interval separating these assessments. Laboratory measurements were completed on a single day, whereas field tests were conducted across three separate sessions, spaced 48 h apart. The testing protocol spanned one week and was carried out both before (pre-intervention) and after (post-intervention) an 8-week training program. A timeline summarizing the sequence of pre- and post-intervention testing procedures is presented in Fig. 1. To ensure consistency, all assessments were conducted at similar times of day for both pre- and post-intervention testing sessions. All field-based assessments were conducted on the same outdoor track under similar environmental conditions, including weather conditions, with testing performed at similar times of day to minimize external variability. Participants were instructed to refrain from engaging in strenuous physical activity for at least 48 h prior to the testing sessions. They were also requested not to consume caffeinated drinks for at least 12 h and to refrain from eating heavy meals for at least 3 h prior to the assessments. This standardized testing protocol was designed to minimize potential confounding variables and ensure reliable and reproducible measurements of the intervention’s effects.

### Laboratory measurements

**CPK enzyme activity measurement**: Participants reported to the laboratory at 9:00 AM. Following a standardized 10-minute low-intensity warm-up consisting of light jogging, dynamic stretching, and preparatory jumps, participants performed a maximum 15-second step test on a 40-cm step (Adams, 1994). The step-test protocol was selected because it imposes a high anaerobic demand and provides a standardized laboratory-based stimulus for activation of the phosphagen energy system. Immediately after completing the step test, a three mL venous blood sample was drawn from the antecubital vein using Ethylenediaminetetraacetic Acid (EDTA)-treated collection tubes. The collected samples were then centrifuged at 3,000 rpm for 10 min at 4 °C in order to separate the plasma fraction. The plasma was carefully aliquoted and preserved at −80 °C until further analysis. Plasma creatine phosphokinase (CPK) levels were later measured using an automated clinical chemistry analyzer (cobas c 111 clinical chemistry analyzer; Roche Diagnostics). The obtained values were reported in units per liter (U/L) (Callegari et al., 2017; Rahmanian et al., 2022).

### Field tests

Participants were evaluated across three separate testing sessions, each separated by a 48-hour interval, to assess various sprint performance variables. The testing sessions were organized as follows: Session 1 involved a 0–40 m sprint test to assess short-distance acceleration and maximum sprint velocity. Session 2 consisted of a 6 × 30 m repeated sprint ability test (RSAT 6 × 30) to evaluate sprint endurance and recovery capacity. Session 3 included a 100-m sprint test to measure maximum sprint performance over a standard competitive distance. The order of testing sessions was standardized for all participants to ensure consistency between pre- and post-intervention assessments, while the 48-hour interval between sessions helped minimize potential fatigue and carry-over effects. Prior to each testing session, participants completed a standardized 10-minute warm-up composed of low-intensity activities, including light jogging and dynamic stretching exercises, to prepare the body and reduce the risk of injury. The tests were carried out as follows:

The 0–40 m sprint test was conducted to evaluate acceleration and maximum sprint velocity. Participants began each trial from a stationary position behind the starting line. Sprint times were recorded at 10-meter intervals (0–10 m, 0–20 m, and 0–30 m) and over the 20–40 m segment, with measurements taken to the nearest 0.01 s using an automated timing system (Bachero-Mena & González-Badillo, 2014). Reliability was confirmed for 0–10 m (ICC = 0.90), 0–20 m (ICC = 0.91), 0–30 m (ICC = 0.89), 0–40 m (ICC = 0.90), and 20–40 m (ICC = 0.91); *p* < 0.001.

RSAT 6 × 30 was conducted to assess anaerobic speed endurance. Participants performed six maximum-intensity 30-meter sprints, starting from a stationary position behind the starting line. Following each sprint, they returned to the opposite end to prepare for the next sprint, which was performed in the reverse direction, with a standardized 20-second passive recovery interval between sprints. Sprint times were recorded to the nearest 0.01 s using an automated timing system. Performance metrics included average sprint time, peak sprint time, total sprint time, and fatigue index. The fatigue index was calculated as:

\begin{eqnarray*}\text{Fatigue Index}~(\%)=100-[(\text{Total Time}/\text{Ideal Time})\times 100] \end{eqnarray*}

where Ideal Time was defined as six times the best sprint time (Hermassi et al., 2017; Megahed et al., 2023; Pyne et al., 2008). Reliability was confirmed for peak sprint time (ICC = 0.89), average sprint time (ICC = 0.91), and fatigue index (ICC = 0.90); *p* < 0.001.

The 100-m sprint performance (100 m SP) was recorded using procedures consistent with the official regulations of the world athletics (World Athletics, 2024). Timing was recorded manually using a Casio stopwatch (Casio, Japan) with an accuracy of 0.01 s, ensuring precise measurement of sprint performance. Although manual timing may be associated with a small measurement error compared with automated timing systems, all 100-m sprint assessments were conducted by experienced officials certified by the national athletics federation. Furthermore, the same timing procedures and evaluators were used consistently during both pre- and post-intervention assessments to ensure comparability of results.

### Training intervention

The training intervention began on June 18, 2024, and extended over an eight-week period, comprising 24 sessions at a frequency of three sessions per week. Each session lasted approximately 75–80 min and was conducted at a consistent time of day to ensure uniformity. All training sessions were conducted on the same outdoor track under similar environmental conditions to minimize environmental variability and ensure consistency throughout the intervention period, and were supervised by the principal investigator and experienced track-and-field coaches who monitored training execution and adherence to the prescribed recovery intervals. Participants were instructed to maintain their usual dietary habits and to refrain from undertaking additional structured training outside the study protocol throughout the intervention period. Each session commenced with a standardized 15-minute low-to-moderate intensity warm-up routine that was identical for all participants incorporating jogging, flexibility exercises, stretching activities, and ABC drills (*e.g.*, high-knee running, butt kicks, and skipping drills) to prepare participants for the subsequent training. The experimental training component followed, lasting 30–35 min. Participants in the CSRG performed a repeated sprint training (RST) program utilizing a CSR protocol, while the TRG followed traditional rest intervals (see Table 2). Both groups followed the same repeated sprint training progression model and exercise intensity framework throughout the intervention period. The primary difference between the groups was the recovery configuration (CSR *versus* traditional rest intervals). Recovery intervals were predetermined and standardized throughout the intervention according to the training protocol. The same repeated sprint training framework was implemented across the three weekly sessions; however, the training load was progressively modified throughout the intervention through planned adjustments in sprint distance, recovery intervals, and exercise intensity, as outlined in Table 2, to ensure an appropriate training stimulus and gradual overload. The RST segment was followed by a 25–30 min session focused on developing sprint-specific skills and technique, including sprint mechanics drills, acceleration drills, start technique practice, stride frequency and stride length drills, and running coordination exercises. The same technical training content was implemented for both groups throughout the intervention period. To conclude, all sessions included a 5-minute cool-down period comprising light stretching and relaxation exercises. This structured training regimen ensured a comprehensive approach to improving sprint performance while maintaining consistency in training delivery across groups.

**Table 2:** Structure of the 8-week RST program for CSRG and TRG.

Weeks	Groups	1st	2nd	3rd	4th	5th	6th	7th	8th
Sprint distances (m) (Main set)	CSRG	2 × 30 m, 2 × 50 m				2 × 30 m, 1 × 50 m, 1 × 60 m			
	TRG	4 × 30 m, 4 × 50 m				4 × 30 m, 2 × 50 m, 2 × 60 m			
Rest between sprints	CSRG	Cluster Rest: 10 s active rest after each sprint + 40 s passive rest after two sprints				Cluster Rest: 15 s active rest after each sprint + 45 s passive rest after two sprints			
	TRG	Traditional Rest: 60 s passive rest				Traditional Rest: 90 s passive rest			
Sets	CSRG	Six sets							
	TRG	Three sets							
Set rest	CSRG	3 minutes							
	TRG	4 minutes							
Intensity %	CSRG	80–85% of		85–90% of		90–95% of		Perform all sprints	
	TRG	maximum effort		maximum effort		maximum effort		at maximum effort	

**Notes.**

CSRG cluster set rest group TRG traditional rest group

For CSRG, cluster-rest intervals were standardized at 10 s of active recovery during weeks 1–4 and 15 s during weeks 5–8; For TRG, rest intervals were standardized according to the protocol throughout the intervention period.

### Statistical analysis

The Shapiro–Wilk test was utilized to evaluate data normality, and Levene’s test was applied to confirm the homogeneity of variance. All variables are summarized using mean values and their corresponding standard deviations (SD). Differences attributable to group, time, and their interaction were analyzed using a mixed-model Analysis of Variance (ANOVA) framework, with factors for group, time, and their interaction (group × time). Bonferroni-corrected *post hoc* analyses were conducted to identify pairwise differences. The effect size for interaction effects was determined using partial eta squared (*η*p^2^), interpreted as minimal (≥0.01), moderate (≥0.06), or large (≥0.14). Changes within each group were further evaluated using Cohen’s d and interpreted as small (0.20 ≤ *d* < 0.50), moderate (0.50 ≤ *d* < 0.80), or large (*d* ≥ 0.80). Percentage change (Δ%) was calculated to describe performance improvements. Reliability estimates were derived from pilot measurements using intraclass correlation coefficients (ICC). A significance level of *p* ≤ 0.05 was adopted for all statistical tests. The statistical analyses were carried out using IBM SPSS Statistics (version 27).

## Results

### Baseline data

At baseline, no significant differences were detected between the two groups across any of the measured variables, with *p*-values ranging from .056 to .421 (*p* > .05). All participants successfully completed the eight-week training intervention, with full adherence reflected in a 100% attendance rate.

### Outcomes of the eight-week intervention

**The effect on CPK enzyme activity:** Analysis of CPK activity revealed a significant group × time interaction (*F* = 55.362; *P* < 0.001; *η*p^2^ = 0.860). Further *post hoc* testing revealed that participants in the CSRG experienced a significantly larger increase in CPK activity compared with those in the TRG.

**Figure 2:**
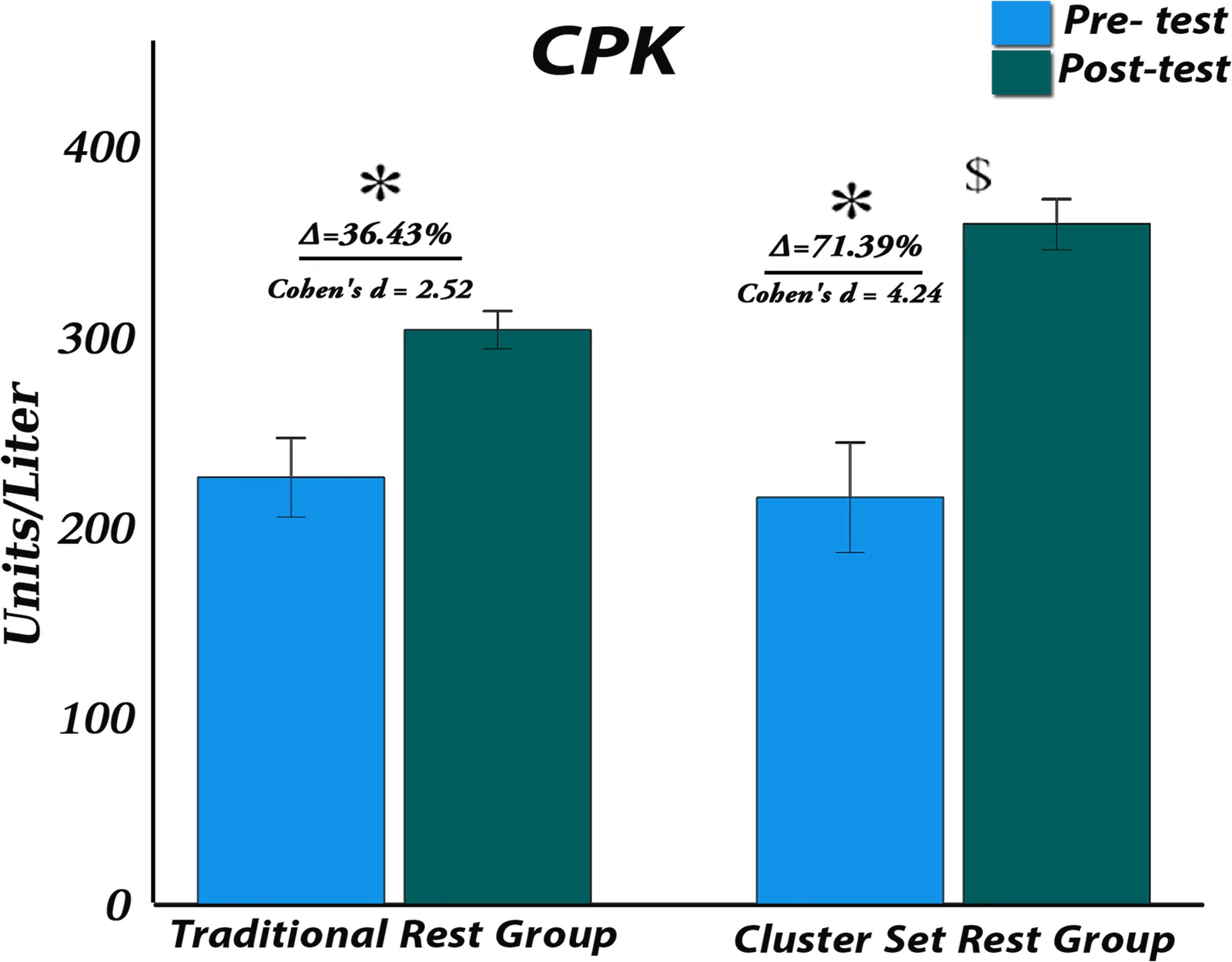
Pre- and post-intervention changes in CPK activity for both groups. Note. CPK, creatine phosphokinase enzyme. $, Significant improvement from the TRG. *, Significant improvement from the pre-test. Δ, Change ratio % from pre-test. *P* < 0.05.

Both groups exhibited significant increases in CPK activity from Pre [(TRG 224.9 ± 29.03 u/l; CSRG 214.4 ± 40.45 u/l), respectively] to Post [(TRG 302.6 ± 13.84 u/l, Δ% = 36.43%, Cohen’s *d* = 2.523, *P* < 0.001; CSRG 358.2 ± 18.74 u/l, Δ% = 71.39%, Cohen’s *d* = 4.238, *P* < 0.001)]. (See Fig. 2)

**The effect on sprint abilities:** Analysis of the 0–40 m sprint test revealed no significant group × time interactions for the 0–10 m (*F* = 2.35, *P* = 0.160), 0–20 m (*F* = 1.617, *P* = 0.235), and 0–30 m (*F* = 1.413, *P* = 0.265) intervals.

In contrast, a significant group × time interaction was identified for the 20–40 m interval (*F* = 11.687, *P* = 0.008, *ηp*^2^ = 0.565), with *post hoc* analyses indicating significantly greater improvements for the CSRG compared to the TRG.

**Figure 3:**
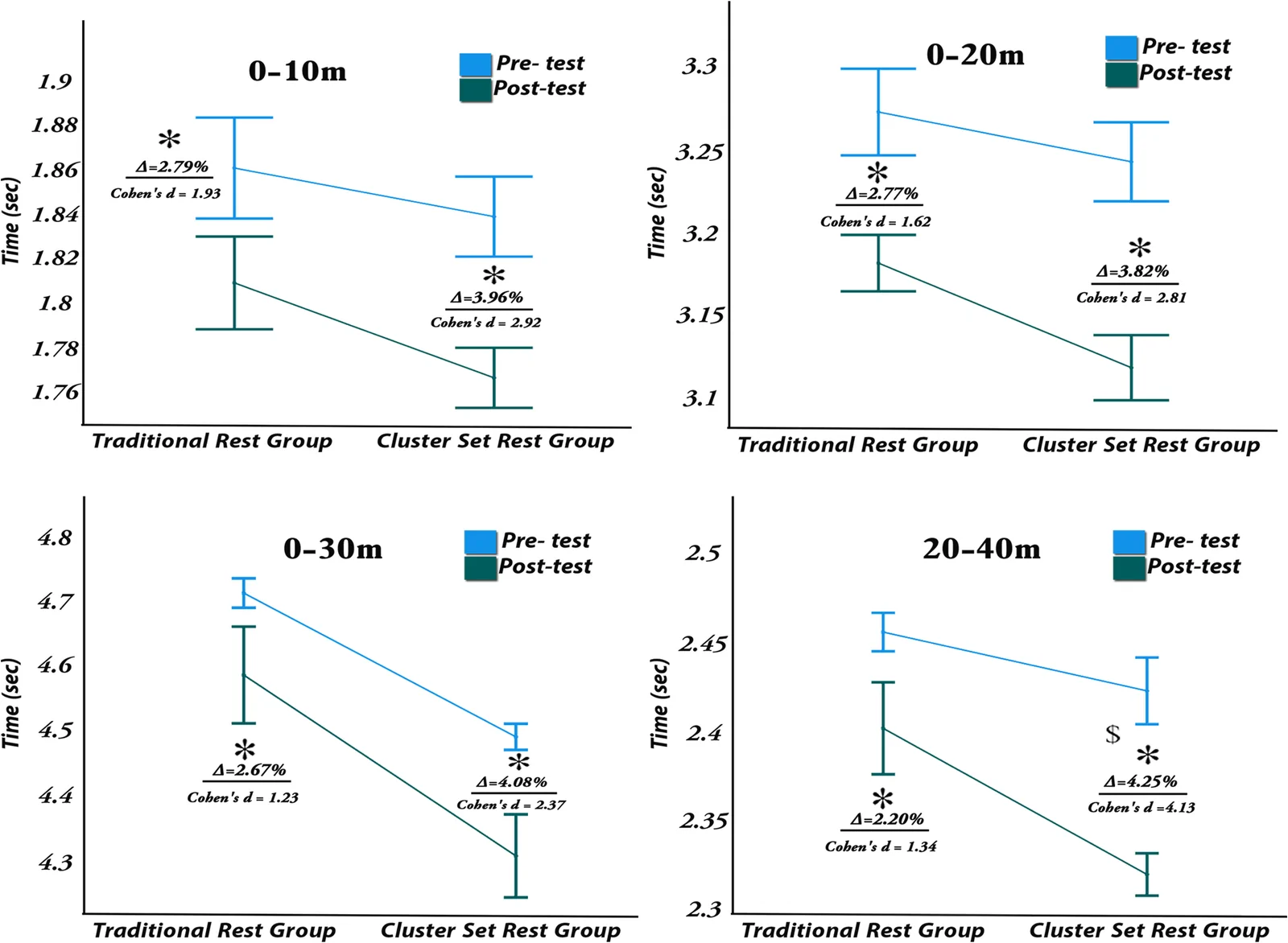
The effect of eight-week training intervention on 0–40 m sprint test. Note. $, Significant improvement from the TRG. *, Significant improvement from the pre-test. Δ, Change ratio % from pre-test. *P* < 0.05.

Both groups demonstrated significant improvements across all measured intervals (0–10 m, 0–20 m, 0–30 m, and 20–40 m) in the 0–40 m sprint test from Pre [(TRG 1.86 ± 0.032 s, 3.27 ± 0.037 s, 4.71 ± 0.032 s and 2.46 ± 0.015 s; CSRG 1.83 ± 0.025 s, 3.24 ± 0.033 s, 4.49 ± 0.028 s and 2.42 ± 0.026 s), respectively] to Post [(TRG 1.81 ± 0.029 s, Δ% = 2.79%, Cohen’s *d* = 1.926, *P* < 0.001, 3.18 ± 0.024 s, Δ% = 2.77%, Cohen’s *d* = 1.629, *P* < 0.001, 4.58 ± 0.104 s, Δ% = 2.67%, Cohen’s *d* = 1.234, *P* = 0.004 and 2.4 ± 0.036 s, Δ% = 2.20%, Cohen’s *d* = 1.339, *P* = 0.002; CSRG 1.77 ± 0.019 s, Δ% = 3.96%, Cohen’s *d* = 2.924, P= <0.001, 3.118 ± 0.027 s, Δ% = 3.82%, Cohen’s *d* = 2.817, *P* < 0.001, 4.31 ± 0.089 s, Δ% = 4.08%, Cohen’s *d* = 2.371, *P* < 0.001 and 2.32 ± 0.017 s, Δ% = 4.25%, Cohen’s *d* = 4.126, *P* < 0.001), respectively] (See Fig. 3).

Analysis of the RSAT 6 × 30 data revealed significant group-by-time interactions across the assessed variable for peak sprinting speed (*F* = 15.45, *P* = 0.003, *ηp*^2^ = 0.632), mean sprinting speed (*F* = 10.649, *P* = 0.010, *ηp*^2^ = 0.542), and fatigue index (*F* = 10.972, *P* = 0.009, *ηp*^2^ = 0.549). Follow-up comparisons showed that participants in the CSRG achieved greater improvements than those in the TRG.

**Figure 4:**
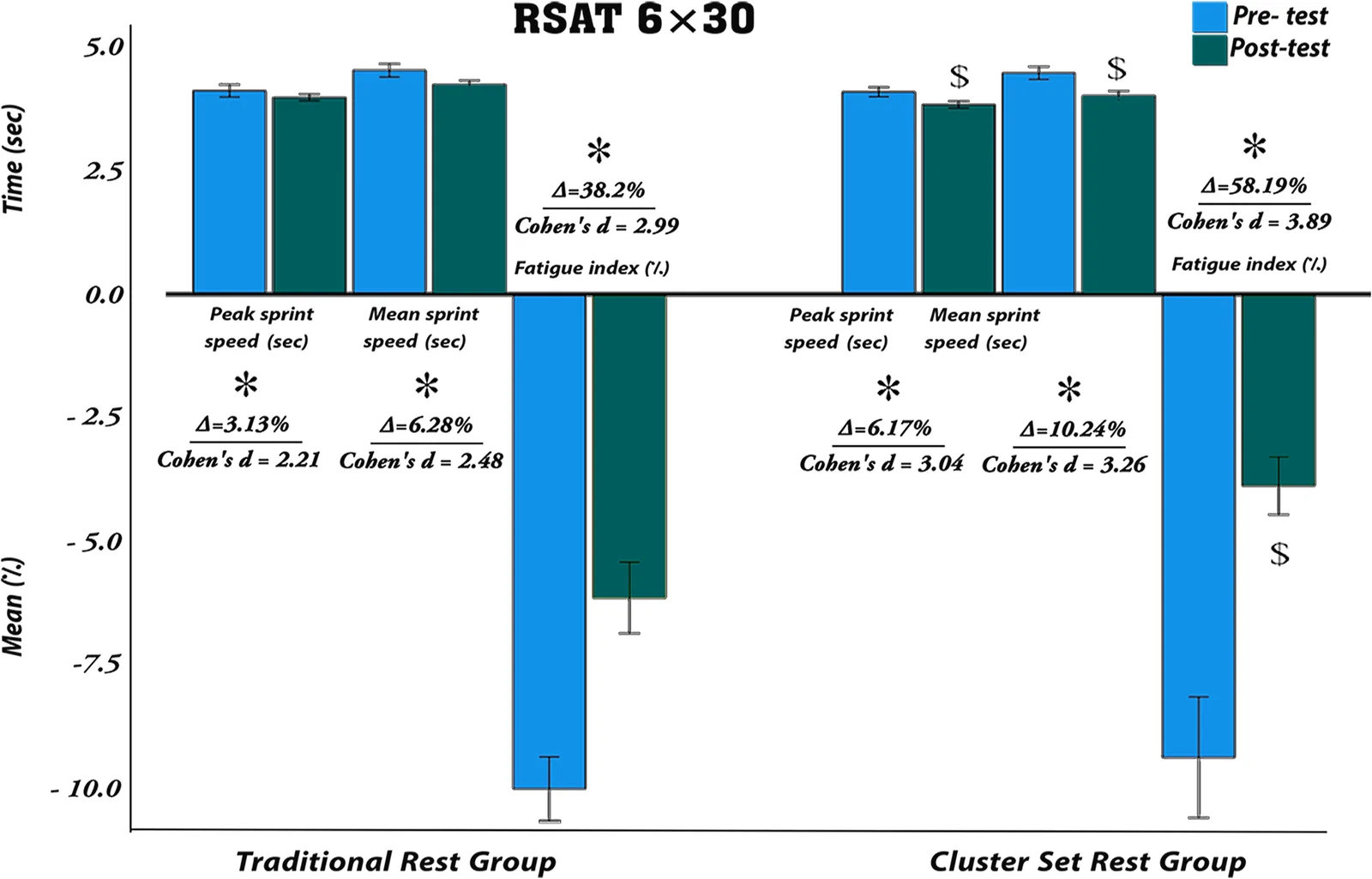
The effect of eight-week training intervention on the RSAT 6  × 30. Note. RSAT 6  × 30 = 6  × 30-m repeated sprint ability test; $, Significant improvement from the TRG. *, Significant improvement from the pre-test. Δ, Change ratio % from pre-test. *P* < 0.05.

Both groups demonstrated significant improvements across all measured parameters (peak sprint speed, mean sprint speed and fatigue index) in the RSAT 6 × 30 from Pre [(TRG 4.12 ± 0.177 s, 4.53 ± 0.189 s and 10.01 ± 0.905%; CSRG 4.09 ± 0.137 s, 4.48 ± 0.179 s and 9.38 ± 1.713%), respectively] to Post [(TRG 3.98 ± 0.092 s, Δ% = 3.13%, Cohen’s *d* = 1.206, *P* < 0.001, 4.24 ± 0.117 s, Δ% = 6.28%, Cohen’s *d* = 2.479, *P* < 0.001 and 6.15 ± 1.012%, Δ% = 38.2%, Cohen’s *d* = 2.99, *P* < 0.001; CSRG 3.84 ± 0.095 s, Δ% = 6.17%, Cohen’s *d* = 3.044, *P* =  < 0.001, 4.02 ± 0.135 s, Δ% = 10.24%, Cohen’s *d* = 3.261, *P* < 0.001 and 3.88 ± 0.818%, Δ% = 58.19%, Cohen’s *d* = 3.891, *P* < 0.001), respectively] (See Fig. 4).

**The effect on 100-m sprint performance:** Analysis of 100-m sprint time (100 m SP time) showed a significant group × time interaction (*F* = 6.475, *P* = 0.031, *ηp*^2^ = 0.418), with *post hoc* analyses indicating significantly greater improvements in the CSRG compared with the TRG.

**Figure 5:**
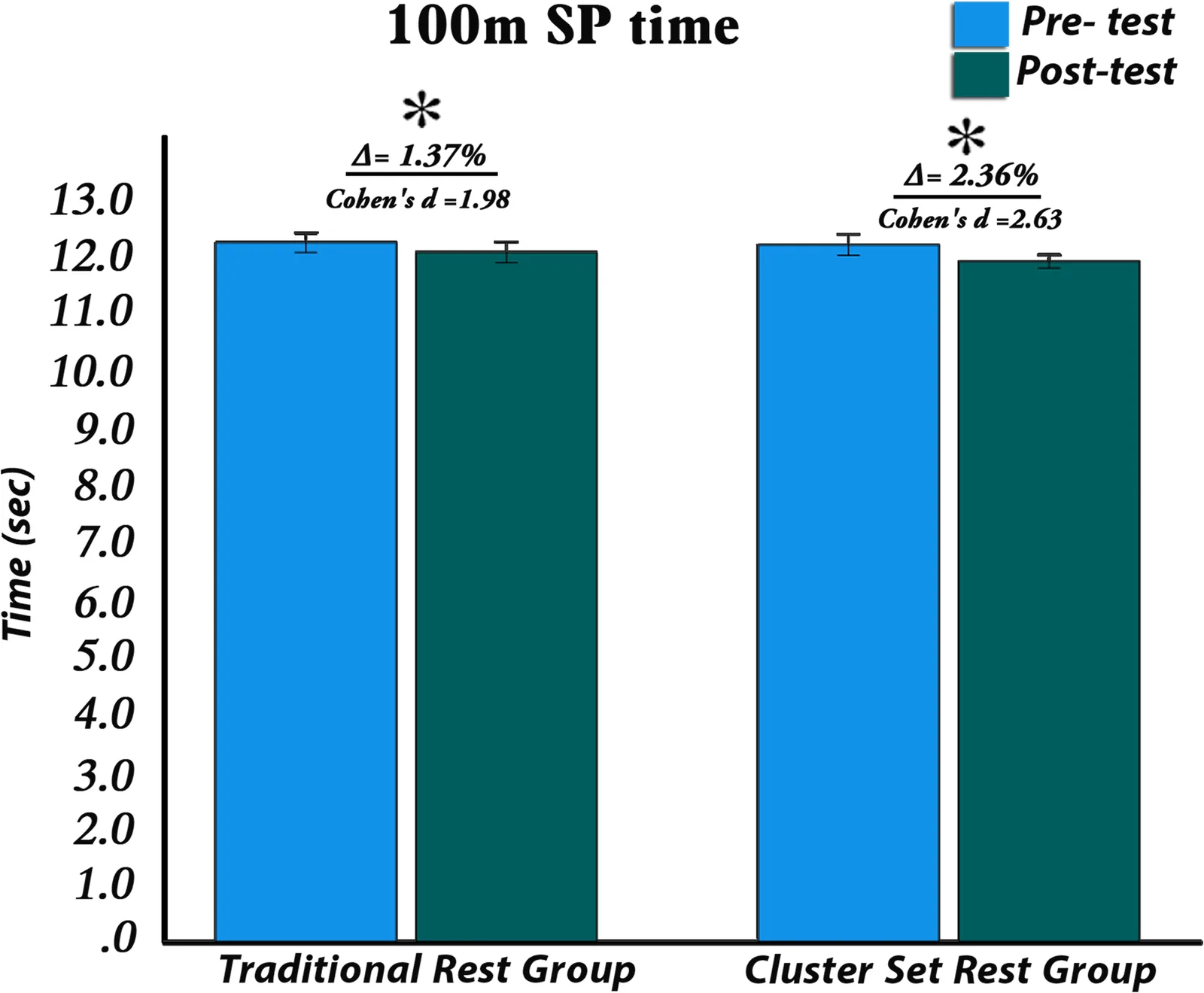
The effect of eight-week training intervention on the 100 m SP. Note. 100 m SP = 100-m sprint performance; $, Significant improvement from the control group. *, Significant improvement from the pre-test. Δ, Change ratio % from pre-test. *P* < 0.05.

Both groups demonstrated significant reductions in 100 m SP time from pre [TRG 12.12 ± 0.236 s; CSRG 12.082 ± 0.252 s] to post [(TRG 11.955 ± 0.248 s; Δ% = 1.37%; Cohen’s *d* = 1.977, *P* < 0.001); (CSRG 11.795 ± 0.16 s; Δ% = 2.36%; Cohen’s *d* = 2.627, *P* < 0.001)] (See Fig. 5).

## Discussion

The present study investigated the comparative effects of CSR *versus* traditional rest interval during an eight-week repeated sprint training program on key physiological and performance metrics, including anaerobic enzyme activity, sprint performance, and 100-m sprint outcomes. The findings demonstrated significant interaction effects favoring the CSR, particularly in terms of enhanced enzymatic activity, improved selected sprint interval performance, and superior repeated sprint ability. These results suggest that the strategic manipulation of intra-set rest intervals can play a crucial role in optimizing physiological adaptations and sprinting capabilities. These results align with literature indicating that more frequent intra-set rest intervals may lead to greater improvements in these performance variables (Api et al., 2023; Cuevas-Aburto et al., 2022; Haff & Harden, 2022; Jukic et al., 2022; Oliver et al., 2015; Ortega-Becerra, Sánchez-Moreno & Pareja-Blanco, 2021; Tufano et al., 2019).

### CPK activity

Regarding CPK enzyme activity, the results demonstrated significant increases in CPK activity in both training groups; however, the improvements were significantly greater in the CSRG compared to the TRG, with a large effect size (*η*p^2^ ≈ 0.86; *P* < 0.001). These findings suggest that the intermittent rest periods incorporated within the cluster-set format may contribute to distinct metabolic and physiological adaptations compared to TRG. The results align with literature indicating that intermittent recovery periods can optimize anaerobic metabolic pathways more effectively than traditional rest structures (Buchheit & Laursen, 2013; Girard, Mendez-Villanueva & Bishop, 2011). The superior enzymatic adaptations observed in the CSRG may be related to the unique physiological demands imposed by cluster-set training. The brief intra-set rest intervals likely facilitated repeated high-intensity efforts while mitigating excessive fatigue, allowing for sustained power output during training sessions (Tufano, Brown & Haff, 2017). This repeated exposure to high-intensity stimuli may have amplified the metabolic adaptations necessary for enhanced anaerobic enzymatic activity (Haff et al., 2008). The significantly greater increases in CPK activity observed in the CSRG (Δ% = 71.39%) compared to the TRG (Δ% = 36.43%) further reinforce the metabolic advantages of CSR. The elevated CPK activity observed in the CSRG may reflect greater phosphocreatine turnover, which is essential for rapid ATP resynthesis during short-duration, high-intensity efforts (Bogdanis et al., 1996). However, CPK is also widely recognized as an indirect marker of muscle damage and exercise-induced muscle stress. Therefore, the observed increases in CPK activity should be interpreted with caution and not exclusively as a positive training adaptation (Baird et al., 2012; Brancaccio, Maffulli & Limongelli, 2007). These findings are consistent with research demonstrating that anaerobic enzymatic activity is significantly higher in trained sprint athletes compared to untrained individuals, reflecting optimal glycolytic adaptations (Hojati et al., 2013). Furthermore, literature have shown that rest periods during intermittent training can significantly impact CPK activity, with prolonged rest intervals leading to lower CPK levels (Ravasi, Aminian & Razaghi, 2006). The cluster-set structure, by allowing brief recovery periods, may have contributed to improved phosphocreatine (PCr) and adenosine triphosphate (ATP) resynthesis while enhancing metabolite clearance (*e.g.*, lactate accumulation) (Oliver et al., 2015). This mechanism could explain the greater metabolic adaptations observed in the CSRG, which likely facilitated the maintenance of velocity across repeated sprint efforts (Gorostiaga et al., 2012). From a practical perspective, the findings suggest that incorporating cluster-set training strategies into sprint training regimens can optimize metabolic adaptations, potentially leading to enhanced sprint performance. The ability to sustain high-intensity efforts with reduced fatigue accumulation is particularly relevant for track and field athletes, where repeated sprinting bouts are a key performance determinant. Future research should explore the long-term effects of cluster-set training on anaerobic capacity, muscle physiology, and sprint performance across different athletic populations. Additionally, investigating the optimal work-to-rest ratios within CSR may provide further insights into maximizing anaerobic metabolic adaptations.

### Sprint performance (0–40 m)

In terms of sprint abilities, our findings indicate that both training regimens elicited significant within-group improvements across all measured sprint intervals (0–10 m, 0–20 m, 0–30 m, and 20–40 m) in the 0–40 m sprint test. However, no significant interaction effects were observed between the groups in the acceleration phases (0–10 m, 0–20 m, and 0–30 m), where sprint velocity increases from a stationary position. This suggests that both training modalities similarly enhanced early-phase sprint mechanics, likely through improvements in lower-limb explosive power and neuromuscular efficiency (Barr & Nolte, 2011). These adaptations may be attributed to improved motor unit recruitment and synchronization, which are critical for initial sprint acceleration (Jukic et al., 2022). In contrast, a significant interaction effect was observed in the flying sprint phase (20–40 m) (*F* = 11.687, *P* = 0.008, *ηp*^2^ = 0.565), indicating that the cluster-set repeated sprint group (CSRG) demonstrated superior improvements in maximum sprint velocity compared to the traditional resistance group (TRG). This finding suggests that cluster-set sprint training may be more effective in sustaining high-velocity sprint performance (Girard, Mendez-Villanueva & Bishop, 2011). These findings align with previous research demonstrating that cluster-set training can enhance maximum sprint velocity performance compared with traditional rest configurations (Asadi & Ramírez-Campillo, 2016; Kenneally, Casado & Santos-Concejero, 2018; Moghadam et al., 2023; Morin et al., 2012; Yilmaz et al., 2021). The underlying mechanisms responsible for these improvements may include increased motor unit recruitment, enhanced muscle power output, and improved acceleration and speed development capabilities lower ground contact times. Likely driven by greater high-speed output during repeated sprint efforts, this may have contributed to the observed performance enhancements (Asadi & Ramírez-Campillo, 2016; De Villarreal et al., 2009). The broader body of evidence strongly supports the use of cluster sets over traditional set configurations when the goal is to maintain sprint velocity (Tufano, Brown & Haff, 2017). However, it is important to acknowledge that many prior studies have compared CSR with traditional set protocols that involved longer continuous work periods and fewer recovery opportunities, which may have resulted in greater fatigue accumulation than CSR (Oliver et al., 2016; Tufano et al., 2016; Tufano et al., 2017). Future research should aim to explore the optimal balance between intra-set recovery and training volume to maximize sprint performance adaptations while minimizing fatigue-induced decrements.

### Repeated Sprint Ability (RSAT 6 × 30)

Additionally, the results of the RSAT 6 × 30 revealed significant interaction effects across peak sprinting speed (*F* = 15.45, *P* = 0.003, *ηp*^2^ = 0.632), mean sprinting speed (*F* = 10.649, *P* = 0.010, *ηp*^2^ = 0.542), and fatigue index (*F* = 10.972, *P* = 0.009, *ηp*^2^ = 0.549). *post hoc* analyses indicated that the CSRG exhibited significantly greater improvements in all three parameters compared to the TRG. These findings support the notion that CSR enhances repeated sprint performance by facilitating energy-system recovery and reducing performance decrements during repeated sprint efforts (Bishop, Girard & Mendez-Villanueva, 2011; Dupont et al., 2010; Girard, Mendez-Villanueva & Bishop, 2011). The significant reductions in fatigue index observed in the CSRG group (Δ% = 58.19%, *P* < 0.001) compared to the TRG (Δ% = 38.2%, *P* < 0.001) underscore the efficacy of interspersed recovery in mitigating performance decrements during repeated sprint tasks. Overall, the findings suggest that while both training interventions effectively improve sprint performance, the implementation of CSR intervals yields superior adaptations in peak sprint speed and sprint fatigue resistance, particularly in the maximum sprint velocity phase of sprinting. Future research should explore the underlying neuromuscular and metabolic mechanisms contributing to these performance enhancements and examine long-term training adaptations to different sprint-specific interventions.

### 100-m sprint performance

As to 100-m sprint performance, the CSRG demonstrated a greater reduction in 100-m sprint time (2.36%) compared with the TRG (1.37%), creating a large effect size between groups (*ηp*^2^ = 0.418). The superior improvements observed in the CSRG may be attributed to the physiological adaptations associated with cluster-set training (Tufano, Brown & Haff, 2017). The greater reduction in 100-m sprint times in the CSRG aligns with literature indicating that incorporating intra-set rest intervals can enhance anaerobic performance by improving motor unit recruitment and reducing peripheral fatigue (Haff et al., 2008). The greater efficiency in force application during ground contact, as suggested by the improved 100-m SP time values, may explain the superior gains observed in the CSRG. Additionally, the higher metabolic stimulus induced by CSR could have contributed to the observed performance improvements through enhanced phosphocreatine resynthesis and glycolytic energy system engagement (Bogdanis et al., 1996; Sahlin, Harris & Hultman, 1979). A particularly noteworthy finding was the significant interaction effect observed in the flying sprint phase (20–40 m), which favored the CSRG over the TRG. This result suggests that the superior improvements in maximum sprint velocity during this phase were a primary contributor to the enhanced 100-m sprint performance in the CSRG. The enhanced ability to sustain higher velocities during the latter stages of the sprint may have been driven by the neuromuscular and metabolic adaptations facilitated by CSR (Cetin, Hindistan & Ozkaya, 2018; Hirvonen et al., 1987). Additionally, the results from the RSAT 6 × 30 revealed significant interaction effects for peak sprinting speed, mean sprinting speed, and fatigue index, all favoring the CSRG compared to the TRG. These findings further support the notion that CSR may enhance repeated sprint performance by preserving sprint mechanics and reducing fatigue-induced decrements (Bishop, Girard & Mendez-Villanueva, 2011; Dupont et al., 2010; Moghadam et al., 2023). Taken together, the results suggest that cluster-set repeated sprint training may represent a useful strategy for improving both maximum sprint velocity and repeated sprint ability, culminating in superior 100-m sprint performance.

### Study limitations

One limitation of the present study is the use of different timing methods across performance assessments. While the 0–40 m sprint and RSAT tests were measured using an automated timing system, the 100-m sprint test was recorded manually. Although the same procedures and evaluators were used consistently throughout the study, a small measurement error associated with manual timing cannot be entirely excluded. Additionally, although efforts were made to control potential confounding variables through standardized training conditions, similar environmental settings, and instructions to avoid additional structured training, the influence of individual differences in training background and daily physical activity outside the study cannot be entirely excluded. Furthermore, the relatively small sample size and the eight-week intervention period may limit the generalizability of the findings and the ability to assess longer-term training adaptations.

### Practical applications

From a practical perspective, coaches and practitioners working with sprint athletes may consider incorporating cluster-set rest configurations within repeated sprint training programs to enhance maximum sprint velocity, repeated sprint ability, and fatigue resistance. The use of brief intra-set recovery periods may help athletes maintain higher training quality, sustain sprint performance, and reduce fatigue accumulation during repeated high-intensity sprint efforts.

## Conclusion

The findings of this study suggest that CSR during repeated sprint training may promote greater physiological and performance adaptations than traditional rest configurations. Specifically, the cluster-set format was associated with greater changes in CPK activity, repeated sprint ability, and selected sprint performance measures compared with traditional rest intervals, with large effect sizes observed across these variables. The metabolic advantages of interspersed recovery periods likely contributed to improved phosphocreatine resynthesis and fatigue resistance, thereby sustaining higher power outputs and optimizing sprint mechanics. These results suggest that cluster-set training may represent a useful strategy for supporting anaerobic adaptations and sprint performance in track and field athletes. Future research should further investigate the long-term impact of cluster-set protocols on neuromuscular and metabolic characteristics, as well as their applicability across different athletic populations. Incorporating cluster-set training into sprint regimens may offer a valuable approach to enhancing competitive sprint performance.

##  Supplemental Information

10.7717/peerj.21689/supp-1 Supplemental Information 1 Raw data from the 0–10 m sprint split, representing acceleration performance during the initial 10 m from a stationary start

10.7717/peerj.21689/supp-2 Supplemental Information 2 Raw data from the 0–20 m sprint split, representing acceleration performance over the first 20 m from a stationary start

10.7717/peerj.21689/supp-3 Supplemental Information 3 Raw data from the 0–30 m sprint split, representing acceleration performance over the first 30 m from a stationary start

10.7717/peerj.21689/supp-4 Supplemental Information 4 Raw data from the 20–40 m sprint split, representing maximal sprint speed performance during the 20–40 m segment

10.7717/peerj.21689/supp-5 Supplemental Information 5 A repeated sprint test (6 × 30 m) to assess anaerobic speed endurance and repeated sprint ability

10.7717/peerj.21689/supp-6 Supplemental Information 6 Measurement of 100-m sprint performance time

10.7717/peerj.21689/supp-7 Supplemental Information 7 Creatine phosphokinase activity

## Additional Information and Declarations

### Data Availability

The following information was supplied regarding data availability: The data is available in the Supplemental Files.
